# Efficacy of leg swing versus quadriceps strengthening exercise among patients with knee osteoarthritis: study protocol for a randomized controlled trial

**DOI:** 10.1186/s13063-022-06282-0

**Published:** 2022-04-18

**Authors:** Ruiyang Li, Pingping Sun, Yu Zhan, Xuetao Xie, Weibing Yan, Congfeng Luo

**Affiliations:** 1 Department of Orthopaedic Surgery, Shanghai Jiao Tong University Affiliated Sixth People’s Hospital, Shanghai, 200233 People’s Republic of China; 2 School of Rehabilitation Science, Shanghai University of Traditional Chinese Medicine, Shanghai, 201203 People’s Republic of China

**Keywords:** Knee osteoarthritis, Physiotherapy, Traditional Chinese exercise, Quadriceps, Strengthening, Home exercise program, Clinical trial, RCT

## Abstract

**Background:**

Knee osteoarthritis (OA) is a leading cause of global disability. According to current guidelines, exercise is the most recommended and important non-surgical treatment for knee OA. However, the best type of exercise for this condition remains unclear. Evidence has shown that traditional Chinese exercises may be more effective. Therefore, the current prospective, two-armed, single-center randomized controlled trial (RCT) aimed to identify an effective physiotherapy for knee OA.

**Methods/design:**

In total, 114 patients with painful knee OA will be recruited from the orthopedic outpatient department of Shanghai Jiao Tong University Affiliated Sixth People’s Hospital. To compare the therapeutic effect of two different home-based exercise programs, the participants will be randomly assigned into the experimental group (leg swing exercise) or the control group (quadriceps strengthening exercise). Each participant in both groups will be required to attend five individual sessions with a physiotherapist who will teach the exercise program and monitor progress. Participants will be instructed to perform the exercises at home every day for 12 weeks. Clinical outcomes will be assessed at baseline and 12 and 24 weeks after starting the intervention. The primary outcomes are average overall knee pain and physical function in daily life. The secondary outcomes include other measures of knee pain, physical function, patient-perceived satisfactory improvement, health-related quality of life, physical activity and performance, muscle strength of the lower limb, and adherence.

**Discussion:**

This study will provide more evidence on the effects of traditional Chinese exercise on improving physical function and relieving joint pain among patients with knee OA. If proven effective, leg swing exercise can be used as a non-surgical treatment for knee OA in the future.

**Trial registration:**

Chinese Clinical Trial Registry ChiCTR2000039005. Registered on 13 October 2020.

**Supplementary Information:**

The online version contains supplementary material available at 10.1186/s13063-022-06282-0.

## Background

Knee osteoarthritis (OA) is a leading cause of global disability [1, 2]. In 2017, the global prevalence of hip and knee OA was approximately 303.1 million cases, and the age-standardized prevalence was 3754.2 per 100,000 [2]. Patients with OA may present with increasing joint pain, worsening physical function, decreased physical activity, and low quality of life [3]. Thus, numerous patients with hip and knee OA require joint replacement surgery, and the economic burden to the society worldwide has been significant [4]. With the aging of the global population and the increasing incidence of obesity, OA can become a serious public health issue.

Currently, there is no definitive treatment for knee OA and effective therapy options are limited [5]. Better treatments are thus needed to relieve OA symptoms and improve physical function while minimizing the economic burden to both patients and the global society [6].

Thus far, exercise is one of the most recommended non-surgical treatments for knee OA regardless of age, disease severity, and comorbidities [5, 6]. Clinical guideline for treating knee OA recommend several types of exercise. These include aquatic exercises, mind-body exercises, and structured land-based exercises such as strength training [6]. Previous studies have found that these non-surgical treatments can relieve pain, improve physical function, enhance quality of life, and delay disease progression to varying degrees [5, 7]. Considering the safety and economic efficiency of exercise for patients with knee OA, lower limb muscle strengthening exercise has become an important part of routine treatment.

In terms of mind-body exercise, traditional Chinese exercises such as T’ai chi and Baduanjin have been shown to have some effects in controlling pain and improving physical function among patients with knee OA [7–10]. Both exercises are a combination of muscle strengthening and aerobic training. However, considering that T’ai chi and Baduanjin are comprehensive whole-body exercises, elderly individuals may find these exercises time-consuming and cognitively demanding. Therefore, a simple exercise program focusing on the comprehensive treatment of the lower limb is required.

In fact, there is another type of traditional Chinese exercise referred to as Daoyin, which is easier and is less cognitively demanding [11]. Although each of these exercises has its own training characteristics, they have common features. In general, Daoyin integrates both static and dynamic exercises with a great emphasis on regulating, breathing, and exercising intrinsic control and mental intent [12].

This study focused on leg swing exercise, which is an extremely important part of Daoyin based on the traditional Chinese medicine theory. This exercise is selected and recommended for three reasons. First, compared with T’ai chi and Baduanjin, the leg swing exercise of Daoyin focuses more on comprehensive training of the lower limb. This exercise is a combination of elements including strength training, active range of motion exercise, and aerobic activity. Second, since patients with knee OA have lower isokinetic strength in the hip muscles than healthy people, strengthening the muscles surrounding the hip joint may decrease pain [13, 14]. Leg swing exercise focuses on the joints of the lower limb including hip and knee joints. Third, as this clinical study will include mostly elderly participants, the exercise program should be simple enough for them to learn so that adherence will be good.

This clinical study aims to identify whether leg swing exercise, which is a type of Chinese traditional exercise, can relieve pain and improve physical function among patients with knee OA. Moreover, it wants to compare the efficacy of two different exercise programs on different outcomes such as knee pain, physical performance, health-related quality of life, patient-perceived satisfactory improvement, and lower limb muscle strength. We hypothesized that participants who take leg swing exercise will have greater improvements in pain intensity and physical function than those who take quadriceps strengthening exercise at 12 and 24 weeks after starting the intervention.

## Methods/design

### Study design

**Fig. 1:**
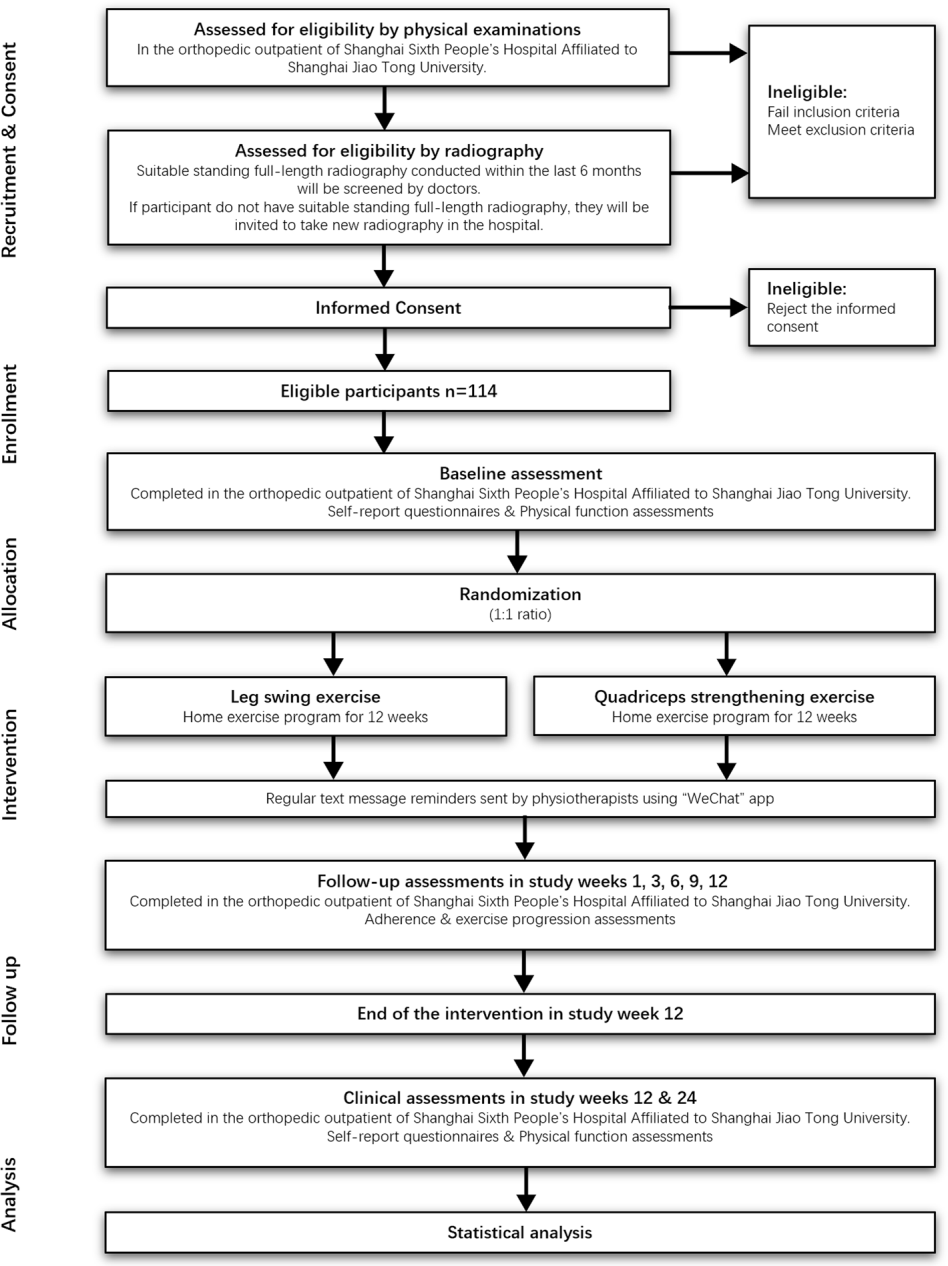
Flow diagram of the RCT

This protocol is designed according to the Standard Protocol Items: Recommendations for Interventional Trials (SPIRIT) guidelines for clinical trials [15] (see Additional file 1) and the Osteoarthritis Research Society International (OARSI) Clinical Trials recommendations for rehabilitation interventions [16]. To the best of our knowledge, previous studies investigated the effects of muscle strengthening exercise in patients with knee OA, focusing mainly on the quadriceps [17]. Leg swing exercise is a novel rehabilitation treatment for knee OA. Meanwhile, the quadriceps strengthening exercise is the established intervention that can validate the efficacy of leg swing exercise [16]. This study will be a prospective, two-armed, single-center randomized controlled trial (RCT) in which participants with painful knee OA will be randomly assigned into the experimental group (leg swing exercise) or the control group (quadriceps strengthening exercise). Figure 1 shows the RCT procedure. The 2–3 year trial will be conducted at Shanghai Jiao Tong University Affiliated Sixth People’s Hospital. Clinical assessments will be performed at baseline and 12 and 24 weeks after starting the intervention. Assessment outcomes will be analyzed in randomization groups. The trial was registered with the Chinese Clinical Trial Registry (ChiCTR2000039005) on October 13, 2020. This study has been approved by the Human Research Ethics Committee of Shanghai Sixth People’s Hospital prior to recruitment, and all participants will provide informed consent.

### Participants

In total, 114 patients aged ≥ 50 years with painful knee OA will be recruited from the orthopedic outpatient department of Shanghai Jiao Tong University Affiliated Sixth People’s Hospital.

The inclusion criteria will be as follows:

1. Patients aged ≥ 50 years old
2. Those with OA in at least one knee according to the American College of Rheumatology Classification Criteria [18]
3. Those with knee pain on most days of the last month
4. Those presenting with knee pain for at least 3 months
5. Those reporting a minimum average overall knee pain intensity of 40 on a 100-mm visual analog score (VAS) for most days in the last month [19]
6. Those with a Kellgren-Lawrence grade (K-L grade) of 2–4 for knee OA
7. Those willing to follow the physiotherapist’s recommendation for exercise training
8. Those with smartphones downloaded with the WeChat app who are willing to receive text message reminders and send feedback to the physiotherapist during the trial.

Knee OA will be further confirmed via physical examinations in the orthopedic outpatient department and a full-length lower limb radiography will be performed in the hospital.

The exclusion criteria will be as follows:

1. Patients with knee joint injection within the last 6 months or planned surgery in the next 12 months
2. Those who have taken oral corticosteroids within the last month or who received intra-articular corticosteroid injection within the last 3 months
3. Those with systemic arthritic conditions
4. Those with previous knee fracture or malignancy
5. Those with a previous history of hip or knee joint replacement, tibial osteotomy, or other surgeries of the hip or knee joint
6. Those with any other neurological, muscular, or joint diseases that may currently affect the physical function of the lower limb
7. Those who have participated in hip or knee muscle strengthening programs within the last 3 months
8. Those without independent mobility
9. Those who reject the leg swing exercise or those who do not provide informed consent.

### Procedures

Eligible patients with knee OA who visit the orthopedic outpatient department of Shanghai Jiao Tong University Affiliated Sixth People’s Hospital will be identified. Primarily, the orthopedic doctor will assess the patient’s eligibility via physical examinations. Then the results of standing full-length radiography conducted within the last 6 months will be screened by an orthopedic doctor. If participants did not undergo radiography, the procedure will be performed in the hospital. After eligibility assessment and the collection of informed consent, the doctor will arrange for a meeting with the treating physiotherapist.

For participants with bilateral symptoms, both knees will be the subjects of rehabilitation. However, only the most symptomatic eligible knee (as identified according to K-L grade and pain intensity) will be selected for assessment. In cases in which bilateral knees are equally symptomatic, the right knee will be nominated.

Participants will visit the same physiotherapist five times (study weeks 1, 3, 6, 9, and 12) over the 12-week intervention and one time 12 weeks after the end of the intervention (study week 24). Baseline and follow-up assessments will be completed at the orthopedic outpatient department of Shanghai Sixth People’s Hospital. At other times, participants will be required to maintain contact with the physiotherapist. Considering that WeChat is the most common mobile social application in China and it can be easily used by elderly individuals, regular text message reminders will be sent by the physiotherapist via this application to help improve adherence. In addition, the physiotherapist can screen participants via video calls through WeChat if necessary.

During each visit, the physiotherapist will assess the adherence of participants and the progression of the home exercise program. Moreover, they will adjust the exercise program according to each participant’s progress and physical condition using the modified Borg Rating of Perceived Exertion (RPE) CR-10 scale [20, 21]. Each individual session will last about one hour. If participants show poor adherence, the causes will be analyzed in the final statistics. Clinical outcomes such as self-reported questionnaire results, physical performance, lower limb muscle strength, and adherence will be assessed 12 and 24 weeks after starting the intervention.

### Data collection and management

Case report forms will be created by the physiotherapist to collect all data obtained via paper-based questionnaires and physical performance tests in the orthopedic outpatient department. The text messages and video records will be stored electronically by the treating physiotherapist. All data will then be transferred and stored in secure electronic databases. Only the statisticians that perform the statistical analyses will have access to the database during the final analysis.

### Randomization allocation concealment

Patients will be recruited in strict accordance with the inclusion and exclusion criteria and will be subsequently randomized into the experimental group (leg swing exercise) or the control group (quadriceps strengthening exercise) at a ratio of 1:1. Specifically, an independent researcher who is not a part of the trial will generate 114 unique seven-digit codes using the random number function in the SAS software version 9.4 (SAS Institute Inc., Cary, NC, USA). Then these codes will be randomized into two groups and will be placed in a sealed, sequentially numbered, opaque envelope [22]. The envelopes will be stored securely. The recruited participants will be assigned to either the experimental or control group by extracting codes from the envelopes.

### Blinding

Considering that most participants may have learned before the clinical trial that quadriceps strengthening exercise is a treatment for knee OA and that the group allocation can be revealed based on the interventional exercises, the participants will not be blinded to the treatment [16]. However, they will be blinded to the study hypotheses. As questionnaire-based outcomes are self-reported and participants are not blinded to treatments, the assessors of questionnaires will not be blinded. Since the physical performance tests are held in other enclosed rooms, assessors who collect physical function outcomes will be blinded. Statisticians who perform statistical analyses will be blinded. If there will be any unexpected adverse event caused by the exercise program, randomization allocation can be immediately unblinded by the study oversight committee.

### Interventions

Both the leg swing exercise and quadriceps strengthening exercise groups will receive group-based exercise training in the orthopedic outpatient department. The physiotherapist will assess the range of motion and the consistency of movement during follow-up visits. Then the intensity of the exercise program will be adjusted according to each participant’s physical condition. Participants will be required to perform exercise at home every day for 12 weeks. All participants are not allowed to perform any additional exercise program for the treatment of knee OA during the clinical trial.

#### Experimental group

**Fig. 2:**
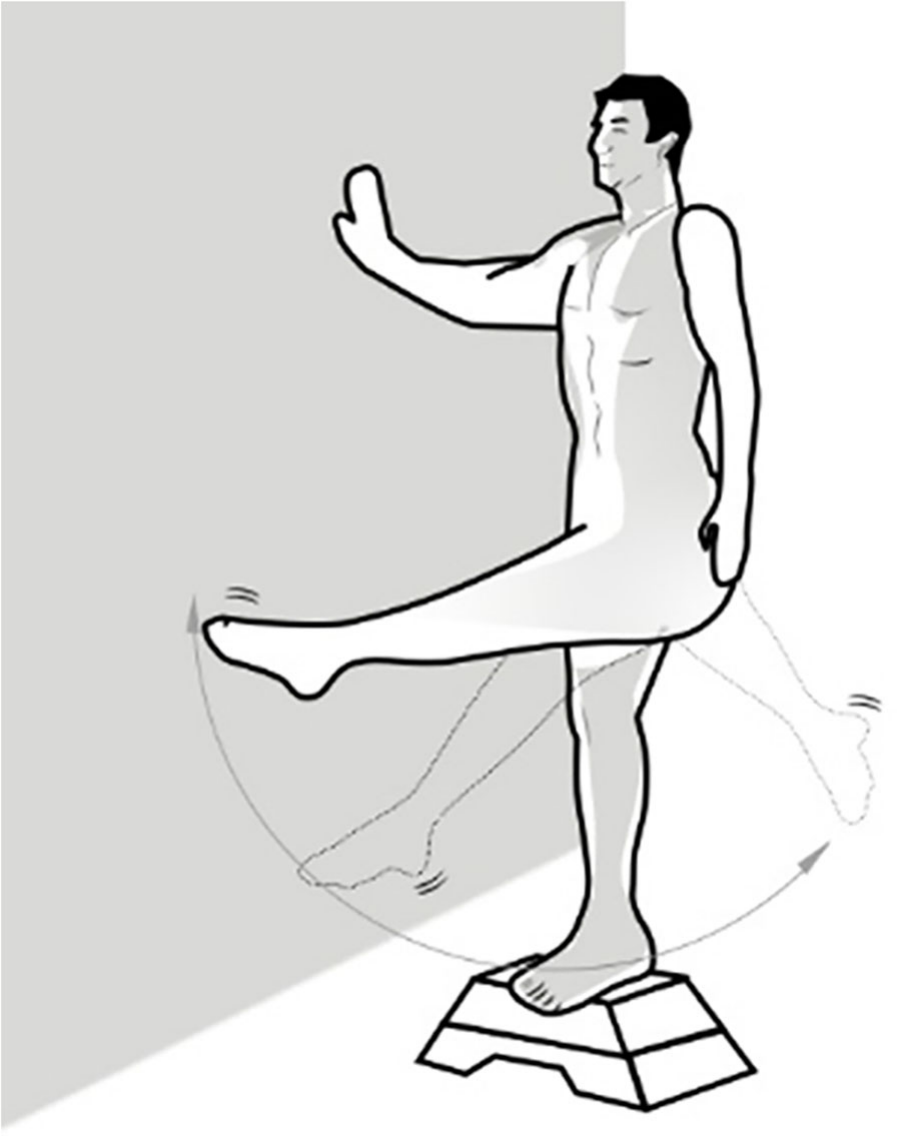
Diagram of the leg swing exercise

The experimental group will receive leg swing exercise training. That is, participants will be instructed to swing the affected leg. To begin the exercise, participants should stand on a platform approximately 10 cm above the ground (Fig. 2). With the unaffected leg standing on the edge of the platform, the affected side of the lower limb will be free in the air. Then the physiotherapist will instruct participants to raise the affected leg to about 45° from the vertical line and to swing the leg to the back of the participants. During exercise, participants should fully relax, thereby adjusting their breathing rhythm, raising the tongue against the palate, and closing their mouth based on the Chinese traditional medicine theory. Once participants are familiar with the whole exercise, the physiotherapist will encourage them to swing the leg to about 60° from the vertical line. Simultaneously, participants must use one or two sides of the handrails to maintain balance. Then they will be required to swing their leg approximately 1000 times (back and forth = one time) per day [11]. The adjustment of training intensity will be based on the physiotherapist’s discretion during follow-up visits to fit each individual. If participants find it difficult to stand on one leg for quite some time, they are encouraged to train the alternative leg and take interval breaks during the whole exercise program. Thus, training time can be extended appropriately.

#### Control group

Participants in the control group will receive quadriceps strengthening exercise training. The specific type of exercise program is straight leg raise. To begin the exercise, participants must lie in supine position and keep their legs straight with a weight attached around the ankle of the affected limb. Meanwhile, they should bend the good limb and make sure both the kneecap and toes of the affected limb are pointing toward the ceiling. Afterwards, participants will be required to slowly raise the affected limb straight up until it gets to about 25–30 cm away from the bed. They should stay in this position for 10 s and then slowly lay down over about 3 s [23, 24]. The participants will be required to perform the exercise as a set of 10 repetitions, for 10 sets each time twice a day [24, 25]. The attached weight for the quadriceps strengthening exercise will be determined by asking participants their level of effort, which should be 5–8 out of 10 (hard to very hard) on the modified Borg RPE CR-10 scale [20, 21].

Participants in both groups will have to visit the physiotherapist at the orthopedic outpatient department of Shanghai Jiao Tong University Affiliated Sixth People’s Hospital for an individual session six times within the next 24 weeks.

### Patient and public involvement

Patients are not involved in recruitment. Moreover, the authors developed the research questions and designed the outcome measures without the help of patients and the public. We asked eight patients with knee OA to perform leg swing and straight leg raise exercises to test the safety of the interventions and the feasibility of the study design. The intensity of interventions was modified according to physical performance and feedback provided by the patients. We adjusted the week 1 plan of the 12-week exercise program to help participants adapt to the intensity of the training as quickly as possible. The physiotherapist will keep in contact with the patients through WeChat and instruct them to gradually increase the amount of exercise performed every day. On the basis of the originally planned outpatient follow-up during study weeks 3, 6, and 9, the week of outpatient follow-up will be added. This will be performed not only to urge patients to do the exercise and improve adherence but also to allow the physiotherapist to confirm that the patients can achieve the required amount of training. The findings of the study will be disseminated to the participants and the public.

### Outcome measures

Table 1 shows the outcomes and the time points for assessment. The primary outcomes are person-centered, reliable, and valid measures recommended for knee OA trials [16, 26].

**Table 1:** Schedule for data collection

Assessments	Baseline	Home exercise program	Follow-up	
		Weeks 1, 3, 6, 9	Week 12	Week 24
Descriptive data	√			
Imaging (K-L grade, HKA angle, JLCA)	√			√
**Adherence**				
Adherence (11-point NRS)		√	√	
Adherence (EARS)		√	√	
Physiotherapy sessions attended (outpatient records)		√	√	
Adherence (WeChat app records)		√	√	
**Primary outcomes**				
Average knee pain in the past week (VAS)	√		√	√
Physical function in the past week (WOMAC)	√		√	√
**Secondary outcomes**				
Average knee pain upon walking in the past week (VAS)	√		√	√
Pain (KOOS)	√		√	√
Other symptoms (KOOS)	√		√	√
Sport and recreational activities (KOOS)	√		√	√
Knee-related quality of life (KOOS)	√		√	√
Improvement in pain (PPSI)			√	√
Improvement in physical function (PPSI)			√	√
Improvement in general (PPSI)			√	√
Health-related quality of life (AQoL 6D)	√		√	√
Physical activity (PASE)	√		√	√
Physical performance (6-step Stair-Climb and Descent Test)	√		√	√
Physical performance (five times sit to stand test)	√		√	√
Physical performance (Stand on one leg test)	√		√	√
Physical performance (40-m fast-paced walk test)	√		√	√
Physical performance (four square step test)	√		√	√
Muscle strength (knee extensors)	√		√	√
Muscle strength (knee flexors)	√		√	√
Muscle strength (hip abductors)	√		√	√
**Additional measures**				
Use of medication	√	√	√	√
Adverse events		√	√	√

#### Descriptive data

Descriptive data include age, sex, duration of knee OA symptoms, affected side of knee OA, previous treatments for knee and other joints, history of other chronic diseases, current use of medication, history of diseases in other joints, current employment status, and expectation of treatment outcome. Information will be collected at baseline in the orthopedic outpatient department. Height and body mass will be evaluated in the outpatient department and body mass index will then be calculated.

#### Imaging outcomes

Kellgren-Lawrence grade (K-L grade) [27], hip-knee-ankle (HKA) angle, and joint line convergence angle (JLCA) will be assessed via standing full-length radiography conducted by the orthopedic doctor. Radiography will be performed again during follow-up visits 24 weeks after starting the intervention.

#### Primary outcomes

The primary outcomes are average overall knee pain assessed using the visual analog score (VAS) and physical function in daily life assessed using the Western Ontario and McMaster Universities (WOMAC) Osteoarthritis Index (Likert version 3.1).

##### Overall knee pain

The average overall knee pain intensity in the last week will be self-reported by participants using the 100-mm VAS, with terminal descriptors of “no pain” (score 0) and “worst pain imaginable” (score 10) [19].

##### Physical function

The WOMAC Osteoarthritis Index (Likert version 3.1) [28] will be used to assess the physical function of participants in daily life within the last week. This index is a self-reported, disease-specific instrument with high reliability, validity, and responsiveness [29]. The daily physical function subscale contains 17 questions, which include most physical activities in social life. These questions are with Likert response options ranging from “no problems” (score 0) to “extreme problems” (score 4), and the total score ranges from 0 to 68, with higher scores indicating worse physical function. The WOMAC physical function subscale has been included in the Knee injury and Osteoarthritis Outcome Score (KOOS) [30, 31] questionnaire.

#### Secondary outcomes

The secondary outcomes include average knee pain upon walking; KOOS; Patient-Perceived Satisfactory Improvement (PPSI); Assessment of Quality of Life 6-Dimension (AQoL-6D) and Physical Activity Scale for the Elderly scores (PASE); 6-Step Stair-Climb and Descent Test, Five Times Sit To Stand Test (FTSST), Stand on One Leg Test, 40-m Fast-Paced Walk Test, and Four Square Step Test results; muscle strength of lower limb; and adherence.

##### Walking pain

The average knee pain intensity upon walking within the last week will be self-reported by participants using the 100-mm VAS with terminal descriptors of “no pain” (score 0) and “worst pain imaginable” (score 10) [19].

##### Knee injury and osteoarthritis outcome score

Other than the assessment of physical function in daily life (WOMAC), the KOOS questionnaire includes other four subscales: pain (nine questions), other symptoms (seven questions), function in sport and recreation (five questions), and knee-related quality of life (four questions) [30, 31]. Every subscale is scored separately with Likert response options from “no problems” (score 0) to “extreme problems” (score 4). The sum of the questions will be calculated. Then the scores of each part will be transformed to a 0–100 scale, with 0 indicating extreme knee problems and 100 indicating no knee problems.

##### Patient-perceived satisfactory improvement

PPSI is a clinically relevant and stable concept for assessing truly meaningful improvements among patients with knee OA. In this study, changes in knee pain, physical function, and overall condition since the start of the intervention will be self-reported by participants using 7-point Likert scales. The terminal descriptors range from “very much worse” to “very much improved” [32]. Participants reporting that they are “much improved” or “very much improved” will be classified as clinically improved.

##### Health-related quality of life

The AQoL-6D contains 20 items that cover six domains including independent living, mental health, relationships, coping, pain and senses [33, 34]. The AQoL utility score ranges from -0.04 (health state worse than death) to 0.00 (death) and 1.00 (completely healthy) with higher scores indicating a better quality of life. AQoL-6D has been previously used in evaluating the impact of an exercise-based intervention on quality of life among patients with OA [24, 35]. This questionnaire is with low respondent burden, low administrator burden, high psychometric properties, and high responsive level [34].

##### Physical activity

Physical activity assessment will be performed using the PASE [36]. This questionnaire assesses both the level and type of recreational and occupational physical activity undertaken by participants within the last week.

##### Physical performance

Six-step Stair-Climb and Descent Test

Participants will be required to ascend and descend six stairs as fast as possible. The use of one handrail is permitted if required [37]. The time taken to finish the test will be recorded. A shorter time to complete the test indicates a greater physical function.

Five Times Sit to Stand Test

In this test, participants sit on an armless chair with their backs resting against it. Then, they are instructed to stand up and sit down without touching the chair’s arms and back. The time taken to complete five repetitions (up and down = one repetition) will be recorded [37, 38]. A shorter time to complete the test indicates a greater physical function.

Stand on One Leg Test

Participants try to stand on one leg while looking straight ahead. They will be instructed to keep hands on their hips. The number of seconds they can hold the posture up to a maximum of 30 s will be recorded. Timing is stopped when participants move their hands off the hips or put a foot down [39]. Two attempts will be allowed with the best time taken as the score. A longer time indicates a greater ability to maintain balance.

Forty-meter Fast-paced Walk Test:

Participants will be instructed to walk 4 × 10 m (excluding turns) quickly but safely. Walking aids can be used and the results will be recorded. The total time taken to complete the task will be recorded. Then the walking speed will be calculated in meters per second (m/s) [40]. A higher walking speed indicates a greater ability of walking short distances and controlling balance.

Four Square Step Test

Four squares are created on the ground using four walking sticks. Participants will be instructed to step into each square as fast as possible while facing forward. The time taken to complete a full sequence will be recorded [41]. Two attempts will be allowed with the best time taken as the score. If participants lose balance or contact a stick, they can have one more attempt. A higher score indicates a greater physical function.

##### Muscle strength

The maximum voluntary isometric strength of the knee extensors, knee flexors, and hip abductors will be measured using the Baseline 250-lb 12-0399 Push-Pull Dynamometer (Fabrication Enterprises Inc., USA). Assessors will show participants the movement that should be tested first. Then the participants will be instructed to confirm the movement. For each muscle strength test, after a warm-up comprising one submaximal and one maximal contractions, participants must perform three consecutive maximal muscle contractions. Each contraction should be performed within 3–5 s. Between each measurement, there will be a 1-min break [42].

Knee extensors and knee flexors will be assessed while in sitting position at 90° hip and knee flexion [43]. The dynamometer will be placed on the anterior and posterior surface of the distal tibia 5 cm proximal to the lateral malleolus. The maximum torque reached over three repetitions of 3 s each will then be recorded and normalized to body mass (Nm/kg).

Hip abductors will be measured while in supine position and the hip in neutral abduction. The dynamometer will be placed on the lateral side of the thigh 5 cm proximal to the lateral femoral condyle. The maximum torque reaching over three repetitions of 3 s each will then be recorded and normalized to body mass (Nm/kg) [42, 43].

##### Adherence

Participants’ adherence to the home exercise program will be assessed using three methods. First, an 11-point Numeric Rating Scale (NRS) (from “completely disagree” to “completely agree”) will be used. The participants will rate up to what extent they agree with the following two statements: “I have been doing my exercise sessions every day as recommended” and “For each exercise, I have been doing the number of repetitions recommended.” Second, according to WeChat and outpatient records, the number of prescribed exercise sessions completed within the last week will be measured at specific time points, with scores ranging from 0 to 7. Third, the Exercise Adherence Rating Scale (EARS) Section B will be used [44]. This questionnaire contains 16 questions, and the results will be self-reported by participants using 5-point Likert scales. The terminal descriptors range from “completely agree” (score 0) to “completely disagree” (score 4). The total score ranges from 0 to 64, with higher scores indicating a better adherence. The physiotherapist will contact participants through WeChat and supervise their exercise programs on a daily basis. If an issue hindering adherence occurs, the participant is required to report the issue to the physiotherapist. The physiotherapist will record any details of the issue in the case report form. The physiotherapist will also arrange for an additional follow-up visit as soon as possible to address the issue. All relevant data will be saved for the final analysis.

#### Additional measures

##### Adverse events

Adverse event is defined as any problem caused by the exercise program. Participants can keep in contact with the physiotherapist via WeChat and can report symptoms of discomfort when doing exercises. The physiotherapist will record any details of the adverse event in the case report form during follow-up visits, and all relevant data will be saved. A study oversight committee will monitor and handle any adverse events if deemed necessary by the chief investigator. Any participant who experiences harm from the trial will receive free treatment. In the final analysis, two specialists will confirm these adverse events.

##### Medication use

Participants will be advised to continue with their usual medication during the trial. However, the medication for knee OA should be discontinued 1 week before the next outpatient follow-up. All data about medications, particularly those for knee OA, including dose and frequency, will be recorded in the case report form by the physiotherapist at baseline and follow-up visits. Other cointerventions will be recorded in the self-report questionnaire.

### Sample size calculation

The sample size was calculated based on average overall knee pain (VAS) and physical function in daily life (WOMAC). The minimal clinically important difference to be detected in knee OA is a change of 18 mm on the 100-mm VAS for knee pain [3] and six units for physical function in WOMAC [45]. Therefore, according to a relevant literature [24], the standard deviations (SD) of clinical change are estimated to be 30 mm for knee pain and 12 units for physical function. Meanwhile, to achieve an effect size of at least 0.5 between groups in either pain or function in a two-armed clinical trial with a power of 80% and a significance level at 0.05 (α error), each group will require 51 participants [46]. To allow for a dropout rate of 10% [47, 48], 57 participants in each group and a total of 114 participants will be recruited by the orthopedic outpatient department.

### Statistical analysis

SPSS Statistics 24.0 software (IBM SPSS Inc., Armonk, New York) will be used for all statistical analyses performed by statisticians blinded to the clinical trial. Descriptive data and baseline and follow-up outcome measures will be presented as mean values (standard deviations) for continuous variables, and counts (percentages) for categorical variables. To manage missing data, we will perform a modified intention-to-treat analysis to include all participants. We will test for a difference between the two treatment arms at each time point (12 weeks and 24 weeks). The primary outcomes will be analyzed using mixed-design repeated measures analysis of variance for inter/intra-group differences. Bonferroni’s post hoc test will be applied if there is a significant result. A similar approach will be used to test the secondary outcomes. Non-parametric statistical tests will be used for any data that do not conform to a normal distribution. Categorical variables will be examined using the logistic regression test. To manage the false discovery rate (FDR), the methodology described by Benjamini and Hochberg will be adopted [49]. An FDR-adjusted *p* value of < 0.05 is considered statistically significant.

## Discussion

This prospective, two-armed, single-center RCT aims to identify whether leg swing exercise, a type of traditional Chinese exercise, is effective in decreasing knee OA symptoms compared with quadriceps strengthening exercise. The results of this RCT will provide more evidence on the effects of traditional Chinese exercise on improving physical function and relieving joint pain among patients with knee OA. If proven to be effective, leg swing exercise, which is a new type of traditional Chinese exercise that is safe, cost-effective, and less cognitively demanding, can be included in the non-surgical treatment regimen for knee OA in the future.

## Trial status

The trial is recruiting patients now. Participant recruitment started on April 20, 2021, and is expected to end on April 30, 2022. The trial was registered at the Chinese Clinical Trial Registry (ChiCTR2000039005) on October 13, 2020.

Protocol Version 2.3 Date: 2022/3/6

## Supplementary Information

**Additional file 1.** SPIRIT 2013 Checklist: Recommendations for Interventional Trials.

## Data Availability

The protocol manuscript does not contain any data, so currently, no additional data are available. The datasets generated and analyzed during the trial will be available in the Figshare repository.

## Abbreviations

AQoL-6D: Assessment of Quality of Life 6-Dimension
Borg RPE: Borg Rating of Perceived Exertion
FDR: False discovery rate
FTSST: Five Times Sit to Stand Test
HKA angle: Hip–knee–ankle angle
K-L grade: Kellgren–Lawrence grade
KOOS: Knee Injury and Osteoarthritis Outcome Score
OA: Osteoarthritis
RCT: Randomized controlled trial
VAS: Visual analog scale
WOMAC: Western Ontario and McMaster Universities Osteoarthritis Index

## References

[1] GBD (2018). 2017 Disease and Injury Incidence and Prevalence Collaborators. Global, regional, and national incidence, prevalence, and years lived with disability for 354 diseases and injuries for 195 countries and territories, 1990-2017: a systematic analysis for the Global Burden of Disease Study 2017. Lancet.

[2] Safiri S, Kolahi AA, Smith E, Hill C, Bettampadi D, Mansournia MA (2020). Global, regional and national burden of osteoarthritis 1990-2017: a systematic analysis of the Global Burden of Disease Study 2017. Ann Rheum Dis.

[3] Bellamy N, Kean WF, Buchanan WW, Gerecz-Simon E, Campbell J (1992). Double blind randomized controlled trial of sodium meclofenamate (Meclomen) and diclofenac sodium (Voltaren): post validation reapplication of the WOMAC Osteoarthritis Index. J Rheumatol.

[4] Salmon JH, Rat AC, Sellam J, Michel M, Eschard JP, Guillemin F (2016). Economic impact of lower-limb osteoarthritis worldwide: a systematic review of cost-of-illness studies. Osteoarthr Cartil.

[5] Wellsandt E, Golightly Y (2018). Exercise in the management of knee and hip osteoarthritis. Curr Opin Rheumatol.

[6] Bannuru RR, Osani MC, Vaysbrot EE, Arden NK, Bennell K, Bierma-Zeinstra SMA (2019). OARSI guidelines for the non-surgical management of knee, hip, and polyarticular osteoarthritis. Osteoarthr Cartil.

[7] Goh SL, Persson MSM, Stocks J, Hou Y, Welton NJ, Lin J (2019). Relative efficacy of different exercises for pain, function, performance and quality of life in knee and hip osteoarthritis: Systematic review and network meta-analysis. Sports Med.

[8] Kang JW, Lee MS, Posadzki P, Ernst E (2011). T'ai chi for the treatment of osteoarthritis: a systematic review and meta-analysis. BMJ Open.

[9] Zou L, Pan Z, Yeung A, Talwar S, Wang C, Liu Y (2018). A Review Study on the Beneficial Effects of Baduanjin. J Altern Complement Med.

[10] An B, Dai K, Zhu Z, Wang Y, Hao Y, Tang T (2008). Baduanjin alleviates the symptoms of knee osteoarthritis. J Altern Complement Med.

[11] Yan WB (2017). Daoyin of traditional Chinese medicine.

[12] Lin C-Y, Wei T-T, Wang C-C, Chen WC, Wang YM, Tsai SY (2018). Acute physiological and psychological effects of Qigong exercise in older practitioners. Evid Based Complement Alternat Med.

[13] Costa RA, Oliveira LM, Watanabe SH, Jones A, Natour J (2010). Isokinetic assessment of the hip muscles in patients with osteoarthritis of the knee. Clinics (Sao Paulo).

[14] Cliborne AV, Wainner RS, Rhon DI, Judd CD, Fee TT, Matekel RL (2004). Clinical hip tests and a functional squat test in patients with knee osteoarthritis: reliability, prevalence of positive test findings, and short-term response to hip mobilization. J Orthop Sports Phys Ther.

[15] Chan AW, Tetzlaff JM, Altman DG, Laupacis A, Gøtzsche PC, Krleža-Jerić K (2013). SPIRIT 2013 statement: defining standard protocol items for clinical trials. Ann Intern Med.

[16] Fitzgerald GK, Hinman RS, Zeni J, Risberg MA, Snyder-Mackler L, Bennell KL (2015). OARSI Clinical Trials Recommendations: Design and conduct of clinical trials of rehabilitation interventions for osteoarthritis. Osteoarthr Cartil.

[17] Øiestad BE, Juhl CB, Eitzen I, Thorlund JB (2015). Knee extensor muscle weakness is a risk factor for development of knee osteoarthritis. A systematic review and meta-analysis. Osteoarthr Cartil.

[18] Altman R, Asch E, Bloch D, Bole G, Borenstein D, Brandt K (1986). Development of criteria for the classification and reporting of osteoarthritis. Arthritis Rheum.

[19] Farrar JT, Young JP, LaMoreaux L, Werth JL, Poole MR (2001). Clinical importance of changes in chronic pain intensity measured on an 11-point numerical pain rating scale. Pain.

[20] Borg G, Ljunggren G, Ceci R (1985). The increase of perceived exertion, aches and pain in the legs, heart rate and blood lactate during exercise on a bicycle ergometer. Eur J Appl Physiol Occup Physiol.

[21] Day ML, McGuigan MR, Brice G, Foster C (2004). Monitoring exercise intensity during resistance training using the session RPE scale. J Strength Cond Res.

[22] Clark L, Dean A, Mitchell A, Torgerson DJ (2021). Envelope use and reporting in randomized controlled trials: A guide for researchers. Res Methods Med Health Sci.

[23] Xie Y, Zhang C, Jiang W, Huang J, Xu L, Pang G (2018). Quadriceps combined with hip abductor strengthening versus quadriceps strengthening in treating knee osteoarthritis: a study protocol for a randomized controlled trial. BMC Musculoskelet Disord.

[24] Bennell KL, Kyriakides M, Metcalf B, Egerton T, Wrigley TV, Hodges PW (2014). Neuromuscular versus quadriceps strengthening exercise in patients with medial knee osteoarthritis and varus malalignment: a randomized controlled trial. Arthritis Rheumatol.

[25] DeLateur B, Lehmann JF, Warren CG, Stonebridge J, Funita G, Cokelet K (1972). Comparison of effectiveness of isokinetic and isotonic exercise in quadriceps strengthening. Arch Phys Med Rehabil.

[26] Messier SP, Callahan LF, Golightly YM, Keefe FJ (2015). OARSI clinical trials recommendations: design and conduct of clinical trials of lifestyle diet and exercise interventions for osteoarthritis. Osteoarthr Cartil.

[27] Kellgren JH, Lawrence JS (1957). Radiological assessment of osteo-arthrosis. Ann Rheum Dis.

[28] Bellamy N, Buchanan WW, Goldsmith CH, Campbell J, Stitt LW (1988). Validation study of WOMAC: a health status instrument for measuring clinically important patient relevant outcomes to antirheumatic drug therapy in patients with osteoarthritis of the hip or knee. J Rheumatol.

[29] McConnell S, Kolopack P, Davis AM (2001). The Western Ontario and McMaster universities osteoarthritis index (WOMAC): a review of its utility and measurement properties. Arthritis Rheum.

[30] Roos EM, Roos HP, Lohmander LS, Ekdahl C, Beynnon BD. Knee injury and osteoarthritis outcome score (KOOS)--development of a self-administered outcome measure. J Orthop Sports Phys Ther 1998; 28:88-96. 10.2519/jospt.1998.28.2.88 9699158

[31] Roos EM, Lohmander LS (2003). The Knee injury and Osteoarthritis Outcome Score (KOOS): from joint injury to osteoarthritis. Health Qual Life Outcomes.

[32] ten Klooster PM, Drossaers-Bakker KW, Taal E, van de Laar MA (2006). Patient-perceived satisfactory improvement (PPSI): interpreting meaningful change in pain from the patient's perspective. Pain.

[33] Osborne RH, Hawthorne G, Lew EA, Gray LC (2003). Quality of life assessment in the community-dwelling elderly: validation of the assessment of quality of life (AQoL) instrument and comparison with the SF-36. J Clin Epidemiol.

[34] Busija L, Pausenberger E, Haines TP, Haymes S, Buchbinder R, Osborne RH (2011). Adult measures of general health and health-related quality of life: Medical Outcomes Study Short Form 36-Item (SF-36) and Short Form 12-Item (SF-12) Health Surveys, Nottingham Health Profile (NHP), Sickness Impact Profile (SIP), Medical Outcomes Study Short Form 6D (SF-6D), Health Utilities Index Mark 3 (HUI3), Quality of Well-Being Scale (QWB), and Assessment of Quality of Life (AQoL). Arthritis Care Res (Hoboken).

[35] Bennell KL, Hinman RS, Metcalf BR, Buchbinder R, McConnell J, McColl G (2005). Efficacy of physiotherapy management of knee joint osteoarthritis: a randomised, double blind, placebo-controlled trial. Ann Rheum Dis.

[36] Martin KA, Rejeski WJ, Miller ME, James MK, Ettinger WH, Messier SP (1999). Validation of the PASE in older adults with knee pain and physical disability. Med Sci Sports Exerc.

[37] Rejeski WJ, Ettinger WH, Schumaker S, James P, Burns R, Elam JT (1995). Assessing performance-related disability in patients with knee osteoarthritis. Osteoarthr Cartil.

[38] Zhang F, Ferrucci L, Culham E, Metter EJ, Guralnik J, Deshpande N (2013). Performance on five times sit-to-stand task as a predictor of subsequent falls and disability in older persons. J Aging Health.

[39] Franchignoni F, Horak F, Godi M, Nardone A, Giordano A (2010). Using psychometric techniques to improve the Balance Evaluation Systems Test: the mini-BESTest. J Rehabil Med.

[40] Dobson F, Hinman RS, Roos EM, Abbott JH, Stratford P, Davis AM (2013). OARSI recommended performance-based tests to assess physical function in people diagnosed with hip or knee osteoarthritis. Osteoarthr Cartil.

[41] Dite W, Temple VA (2002). A clinical test of stepping and change of direction to identify multiple falling older adults. Arch Phys Med Rehabil.

[42] Pua YH, Wrigley TV, Cowan SM, Bennell KL (2008). Intrarater test-retest reliability of hip range of motion and hip muscle strength measurements in persons with hip osteoarthritis. Arch Phys Med Rehabil.

[43] Buckinx F, Croisier JL, Reginster JY, Dardenne N, Beaudart C, Slomian J, Leonard S (2017). Reliability of muscle strength measures obtained with a hand-held dynamometer in an elderly population. Clin Physiol Funct Imaging.

[44] Newman-Beinart NA, Norton S, Dowling D, Gavriloff D, Vari C, Weinman JA (2017). The development and initial psychometric evaluation of a measure assessing adherence to prescribed exercise: the exercise adherence rating scale (EARS). Physiotherapy.

[45] Tubach F, Ravaud P, Baron G, Falissard B, Logeart I, Bellamy N (2005). Evaluation of clinically relevant changes in patient reported outcomes in knee and hip osteoarthritis: the minimal clinically important improvement. Ann Rheum Dis.

[46] Borm GF, Fransen J, Lemmens WA (2007). A simple sample size formula for analysis of covariance in randomized clinical trials. J Clin Epidemiol.

[47] Gillies K, Kearney A, Keenan C, Treweek S, Hudson J, Brueton VC (2021). Strategies to improve retention in randomised trials. Cochrane Database Syst Rev.

[48] Walters SJ, Bonacho Dos Anjos Henriques-Cadby I, Bortolami O, Flight L, Hind D, Jacques RM, et al. Recruitment and retention of participants in randomised controlled trials: a review of trials funded and published by the United Kingdom Health Technology Assessment Programme. BMJ Open 2017;7:3. 10.1136/bmjopen-2016-015276 PMC5372123 28320800

[49] Benjamini Y, Hochberg Y (1995). Controlling the false discovery rate: a practical and powerful approach to multiple testing. J R Stat Soc Ser B.

